# A multiple hold-out framework for Sparse Partial Least Squares

**DOI:** 10.1016/j.jneumeth.2016.06.011

**Published:** 2016-09-15

**Authors:** João M. Monteiro, Anil Rao, John Shawe-Taylor, Janaina Mourão-Miranda

**Affiliations:** a Department of Computer Science, University College London, London, UK; b Max Planck University College London Centre for Computational Psychiatry and Ageing Research, University College London, London, UK

**Keywords:** Machine learning, Sparse methods, Partial Least Squares, Neuroimaging, Mini-Mental State Examination, Dementia

## Abstract

**Background:**

Supervised classification machine learning algorithms may have limitations when studying brain diseases with heterogeneous populations, as the labels might be unreliable. More exploratory approaches, such as Sparse Partial Least Squares (SPLS), may provide insights into the brain's mechanisms by finding relationships between neuroimaging and clinical/demographic data. The identification of these relationships has the potential to improve the current understanding of disease mechanisms, refine clinical assessment tools, and stratify patients. SPLS finds multivariate associative effects in the data by computing pairs of sparse weight vectors, where each pair is used to remove its corresponding associative effect from the data by matrix deflation, before computing additional pairs.

**New method:**

We propose a novel SPLS framework which selects the adequate number of voxels and clinical variables to describe each associative effect, and tests their reliability by fitting the model to different splits of the data. As a proof of concept, the approach was applied to find associations between grey matter probability maps and individual items of the Mini-Mental State Examination (MMSE) in a clinical sample with various degrees of dementia.

**Results:**

The framework found two statistically significant associative effects between subsets of brain voxels and subsets of the questions/tasks.

**Comparison with existing methods:**

SPLS was compared with its non-sparse version (PLS). The use of projection deflation *versus* a classical PLS deflation was also tested in both PLS and SPLS.

**Conclusions:**

SPLS outperformed PLS, finding statistically significant effects and providing higher correlation values in hold-out data. Moreover, projection deflation provided better results.

## Highlights

• SPLS framework which tests model reliability by fitting it to several data splits.
• Framework was applied to brain anatomy and individual items of the MMSE score.
• The adequate number of voxels and clinical items was selected automatically.
• SPLS found two associative effects between sparse brain voxels and MMSE items.
• Projection deflation provided better results than a classical PLS deflation.

## Graphical abstract

**Figure:**
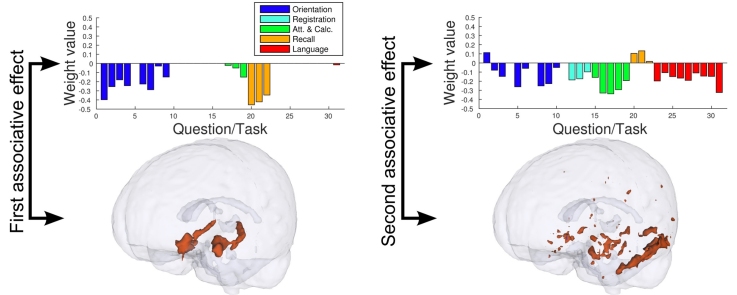

## 1 Introduction

Pattern recognition algorithms have been successfully applied to analyse neuroimaging data for a variety of applications, including the study of neurological and psychiatric diseases. However, so far, most of these studies have focused on supervised binary classification problems, i.e. they summarise the clinical assessment into a single measure (e.g. diagnostic classification) and the output of the models is limited to a probability value and, in most cases, a binary decision (e.g. healthy/patient) (Ecker et al., 2010, Mourão-Miranda et al., 2005, Nouretdinov et al., 2011, Orrù et al., 2012, Rao et al., 2011, Klöppel et al., 2008). This may present itself as a limitation when applying machine learning techniques to study brain diseases whose underlying disease process is not yet completely understood and, therefore, might have an unreliable categorical classification.

More exploratory machine learning approaches, such as Partial Least Squares (PLS), may provide useful insights into the brain's mechanisms by finding relationships between different measures (i.e. views) from the same subjects, more specifically, between neuroimaging and clinical/demographic data in a clinical population. By identifying these relationships, one can improve the current understanding of disease mechanisms. PLS identifies a projection or latent space containing the relevant information in both views, it does this by finding pairs of weight vectors which maximise the covariance between the projections of the two views. By studying this latent space, one can learn about the underlying relationship between clinical information and brain measures. PLS was first introduced to neuroimaging by McIntosh et al., who used PLS to compare encoding and recognition of faces using PET data as one view, and the reaction time of the subjects during a face matching and recognition task as the other view (McIntosh et al., 1996). Since then, it has been applied to neuroimaging data to investigate emotional processing (Keightley et al., 2003), memory (Nyberg et al., 1996, Della-Maggiore et al., 2000), and personality (Keightley et al., 2003). PLS can also be used in a regression framework, i.e. to predict one view from the other. In this context, PLS has been applied to predict the effects of nicotine on behaviour using data from a fMRI task (Giessing et al., 2007), and to analyse the relationship between the shapes of sub-cortical regions using features derived from structural MRI scans (Rao et al., 2008). The technique has also been applied to clinical studies, e.g. to study amyloid deposition in patients with Alzheimer's disease using PET images (Price et al., 2004), and to study the relationships between neuropsychological tests and MRI volume measures in schizophrenia (Nestor et al., 2002). For an extensive review of the applications of PLS, please refer to the paper by Krishnan et al. (2011).

One limitation of these PLS applications is that in high dimensional datasets the interpretability of the models is very difficult, due to the fact that PLS outputs pairs of non-sparse weight vectors, which means that all the features in both views will be used for the model. In order to overcome this issue and to remove noisy features, a variant of PLS called Sparse PLS (SPLS) has been proposed. SPLS selects a subset of important features to be used in the model, which greatly facilitates interpretability. The number of features that are included in the model is controlled by a pair of sparsity hyper-parameters (one for each view). Sparse methods are especially appealing when using high dimensional data, such as brain scans, due to the fact that they automatically select the most relevant voxels for the model. SPLS provides sparse weight vector pairs, where each pair can be seen as a component describing a multivariate associative “effect” in the data. After the algorithm computes a weight vector pair, this is used to deflate the data matrices, i.e. to remove the effect explained by these vectors from the data. The process is then repeated to find the next associative effect.

Some of the earlier applications of SPLS have been in the genetics field, e.g. Lê Cao et al. (2008) and Parkhomenko et al. (2009). These studies proposed SPLS algorithms that are relatively fast, however, the weight computation might not be very stable when the sparsity hyper-parameters are very strict, i.e. very sparse solutions are enforced. Witten et al. (2009) and Witten and Tibshirani (2009) proposed a SPLS algorithm that is slower than the previously mentioned approaches, but avoids this instability (this will be further explained in Section 2.3.1).

While the use of SPLS in neuroimaging is not as extensive as in genetics, there are a few such examples in the literature, e.g. to investigate associations between genetics and fMRI (Le Floch et al., 2012, Lin et al., 2014), structural MRI ROIs and clinical scores (Labus et al., 2015), and structural MRI and Diffusion Tensor Imaging (DTI) (Avants et al., 2010). A closely related method, Sparse Canonical Correlation Analysis (SCCA), has been applied by Avants et al. to investigate correlations between structural MRI data (grey matter) and clinical variables coming from the Philadelphia Brief Assessment of Cognition (PBAC) (Avants et al., 2014). The PBAC test contains 20 variables grouped into 5 psychometric sub-scales, which test different cognitive and behavioral/comportment deficits. The authors fitted 5 models, each one with the MRI scans as one view and the clinical variables of a specific sub-scale as the other view. The results showed that SCCA was able to find relationships between psychometric batteries and grey matter density, claiming to be the first study to have done it (Avants et al., 2014).

Despite the interesting results obtained so far with SPLS and SCCA models, these methods can be computationally demanding when applied to high dimensional data. In order to reduce computational time, previous studies have applied different strategies, including: fixing the level of sparsity instead of finding the optimal level (Avants et al., 2010); using sparsity only in one view (Avants et al., 2014); applying a univariate dimensionally reduction step prior to SPLS (Le Floch et al., 2012); or summarizing the neuroimaging information into brain regions (instead of performing the analysis voxel-wise) (Le Floch et al., 2012, Labus et al., 2015). These strategies rely on *a priori* assumptions regarding data structure and sparsity levels, which might not align well with the true underlying associative effects. In addition, previously proposed SPLS/SCCA algorithms use deflation methods which are inherited from non-sparse methods (PLS or CCA), but might be inappropriate when used with sparse methods.

The use of sparsity in both views is still an issue in SPLS/SCCA models, especially when the statistical significance of the associative effects is tested using permutation tests. If no *a priori* sparsity assumptions are made, the computational time necessary to optimise the sparsity hyper-parameters using a regular nested cross-validation may be too large. In order to make the method computationally feasible, and to check how reliable is the weight vector computation, we propose a new framework which uses multiple hold-out datasets. This framework also applies a different deflation strategy to the ones normally used with SPLS, and allows for each associative effect to have different levels of sparsity on each view.

By studying the effects described by the SPLS weight vector pairs, one can understand the relationships between patterns of brain anatomy and clinical variables in specific patient populations (e.g. dementia). In addition, by projecting the data onto these weights (latent space), one can try to stratify the patients in a less rigid way, and to help refine current clinical assessment tools. Despite our previous application of SPLS to study sparse associations between brain scans and clinical/demographic variables (Monteiro et al., 2015), the use of sparsity on the clinical scores is still often overlooked. Moreover, the clinical scores used are usually summary results of clinical exams. Some of these exams have several questions/tasks covering different areas of cognition, e.g. the Mini-Mental State Examination (MMSE), which is often used in patients with dementia. In some cases, it might be useful to study not how different clinical/demographic variables are associated with the brain, but how the questions/tasks from a specific clinical exam associate with the brain. Such studies might help to refine the current clinical exams, which can be achieved by finding which questions/tasks are associated with changes in brain structure. However, it is also a more challenging problem, since the data encoded in individual questions/tasks is noisier than a summarised clinical exam score.

In this paper, we applied the proposed SPLS framework to a dementia dataset containing structural MRI data as one view, and the scores for each individual MMSE question/task as the other view. As far as we are aware, this is the first study to do so. Sparsity was applied to both views and no *a priori* information about the structure of the data was provided to the model, which distinguishes the present work from previous studies (Avants et al., 2014, Lin et al., 2014). The lack of *a priori* assumptions allows the model to freely look for associations in the data without being constricted by pre-defined brain regions or sub-scales of clinical test scores. This is especially important when the condition being studied still lacks strong evidence to make assumptions regarding behaviour and its relationship with brain structure.

Finally, the performance of both PLS and SPLS was compared using the proposed framework, and two deflation strategies were compared in their ability to generate statistically significant weight vector pairs, and how well they generalise for unseen data.

## 2 Materials and methods

### 2.1 PLS

Let us consider a matrix containing neuroimaging information $\text{X}\in {\mathbb{R}}^{n\times p}$ and a matrix containing clinical information $\text{Y}\in {\mathbb{R}}^{n\times q}$, where *n* is the number of samples, *p* is the number of voxels and *q* is the number of clinical variables. PLS computes a pair of weight vectors **u** and **v**, such that the covariance between the projections of **X** and **Y** onto these weight vectors is maximised (Wegelin, 2000):

(1) $$\begin{array}{l} {\mathrm{maximise}}_{∥\text{u}{∥}_{2}=∥\text{v}{∥}_{2}=1}\;\mathrm{Cov}(\text{Xu},\text{Yv})\\ ={\mathrm{maximise}}_{∥\text{u}{∥}_{2}=∥\text{v}{∥}_{2}=1}\;{\text{u}}^{⊤}{\text{X}}^{⊤}\text{Yv} \end{array}$$

The solution of the problem expressed in Eq. (1) is obtained by the rank-1 approximation of **X**^⊤^**Y** performed using Singular Value Decomposition (SVD) (Lê Cao et al., 2008).

After the first weight vector pair is computed, the effect described by these vectors is removed from the data by matrix deflation, and the process is repeated in order to compute additional weight vector pairs. A brief description of some deflation methods will be presented in Section 2.3.3.

### 2.2 SPLS

In order to understand SPLS, it is important to introduce an older method called Canonical Correlation Analysis (CCA) (Hotelling, 1936), which finds **u**, **v** such that the correlation between the projections **X** and **Y** onto these weight vectors is maximised (Corr(**Xu**, **Yv**)):

(2) $$\begin{array}{c} {\mathrm{maximise}}_{\text{u},\text{v}}\;{\text{u}}^{⊤}{\text{X}}^{⊤}\text{Yv}\\ \mathrm{subject}\;\mathrm{to}\\ {\text{u}}^{⊤}{\text{X}}^{⊤}\text{Xu}⩽1,\;{\text{v}}^{⊤}{\text{Y}}^{⊤}\text{Yv}⩽1 \end{array}$$

Witten et al. proposed a sparse version of CCA, which works by adding *l*_1_-norm constraints to the CCA optimisation problem:

(3) $$\begin{array}{c} {\mathrm{maximise}}_{\text{u},\text{v}}\;{\text{u}}^{⊤}{\text{X}}^{⊤}\text{Yv}\\ \mathrm{subject}\;\mathrm{to}\\ {\text{u}}^{⊤}{\text{X}}^{⊤}\text{Xu}⩽1,\;{\text{v}}^{⊤}{\text{Y}}^{⊤}\text{Yv}⩽1,\;\|\text{u}{\|}_{1}⩽c_{u},\;\|\text{v}{\|}_{1}⩽c_{v} \end{array}$$

However, due to the high dimensionality of the data, the matrices **X^⊤^X** and **Y^⊤^Y** were substituted by identity matrices, which leads to the following optimisation problem (Witten et al., 2009):

(4) $$\begin{array}{c} {\mathrm{maximise}}_{\text{u},\text{v}}\;{\text{u}}^{⊤}{\text{X}}^{⊤}\text{Yv}\\ \mathrm{subject}\;\mathrm{to}\\ \|\text{u}{\|}_{2}^{2}⩽1,\;\|\text{v}{\|}_{2}^{2}⩽1,\;\|\text{u}{\|}_{1}⩽c_{u},\;\|\text{v}{\|}_{1}⩽c_{v} \end{array}$$

where *c*_*u*_ and $c_{v}$ are the regularisation hyper-parameters that control the *l*_1_-norm (∥·∥_1_) constraints of **u** and **v**, respectively. The *l*_1_-norm constraints impose sparsity, which means that the lower the values of *c*_*u*_ and $c_{v}$ are, the stronger the *l*_1_ constraint on the corresponding view is. However, this type of constraint can only select up to *n* features if *p* > *n*, moreover, it will remove features which are relevant for the model, but correlated with other features which are already included. This issue is addressed by the addition of *l*_2_-norm constraints (Zou and Hastie, 2005). In order for both *l*_1_-norm and *l*_2_-norm constraints to be active, the values of the hyper-parameters must be $1⩽c_{u}⩽\sqrt{p}$ and $1⩽c_{v}⩽\sqrt{q}$ (Witten et al., 2009).

This method is referred to as “diagonal penalised CCA” in the original paper (Witten et al., 2009). However, what is being maximised is no longer the correlation between the projections, but the covariance, which makes this problem equivalent to SPLS.

The weight vectors **u** and **v** have the same length as the number of features in the corresponding view, i.e. $\text{u}\in {\mathbb{R}}^{p\times 1}$ and $\text{v}\in {\mathbb{R}}^{q\times 1}$. They represent the latent space found by SPLS, capturing multivariate associative effects between the two views. Since sparsity constraints are applied to the model, several entries of **u** and **v** will be equal to zero. By looking at the paired vectors, one can identify the features in each view related with each associative effect. In summary, each weight vector pair **u** and **v** captures a multivariate associative effect between both views, in the present paper, it will capture an association between a subset of voxels in brain scans and a subset of individual items in a clinical exam (MMSE).

#### 2.2.1 SPLS algorithm

The problem expressed in Eq. (4) is solved by the following algorithm (Witten et al., 2009):

1 Let **C** ← **X^⊤^Y**
2 Initialise **v** to have ∥**v**∥_2_ = 1
3 Repeat until convergence:
  (a) Update **u**:
    i. **u** ← **Cv**
    ii. $\text{u}\leftarrow \frac{S(\text{u},\Delta_{u})}{∥S(\text{u},\Delta_{u}){∥}_{2}}$, where Δ_*u*_ = 0 if this results in ∥**u**∥_1_ ⩽ *c*_*u*_; otherwise, Δ_*u*_ is set to be a positive constant such that ∥**u**∥_1_ = *c*_*u*_.
  (b) Update **v**:
    i. **v** ← **C**^⊤^**u**
    ii. $\text{v}\leftarrow \frac{S(\text{v},\Delta_{v})}{∥S(\text{v},\Delta_{v}){∥}_{2}}$, where $\Delta_{v}=0$ if this results in $∥\text{v}{∥}_{1}⩽c_{v}$; otherwise, $\Delta_{v}$ is set to be a positive constant such that $∥\text{v}{∥}_{1}=c_{v}$.
4 Deflate **C**

where *S*(·, ·) is the soft-thresholding operator defined as *S*(*a*, *λ*) = sgn(*a*)(|*a*| − *λ*)_+_, where *λ* > 0 is a constant and *x*_+_ is equal to *x* if *x* > 0 and *x* = 0 if *x* ⩽ 0 (Witten et al., 2009). The initialisation of **v** in step 2 can be done in several ways (Witten et al., 2009, Parkhomenko et al., 2009, Waaijenborg et al., 2008), in this study, it was done by taking the first component of the SVD of **C** (Witten et al., 2009). Δ_*u*_ and $\Delta_{v}$ have to be set so that the *l*_1_-norm constraints are obeyed. This is done by iteratively searching for Δ_*u*_ and $\Delta_{v}$, such that ∥**u**∥_1_ ≈ *c*_*u*_ and $∥\text{v}{∥}_{1}\approx c_{v}$.

In other SPLS algorithms, the sparsity is set by adjusting *λ* instead of the *l*_1_-norm constraints (Parkhomenko et al., 2009, Lê Cao et al., 2008). This will make the algorithms faster, since Δ_*u*_ and $\Delta_{v}$ do not have to be searched iteratively. However, by setting *λ* directly, there are situations in which this value might be too high and all the entries of **u** or **v** will be set to zero, i.e. no variables are included in the model. The exact value of *λ* for which this happens is dataset dependent. On the other hand, by using *c*_*u*_ or $c_{v}$ to set ∥·∥_1_ = 1, there is a guarantee that at least one entry of the corresponding weight vector will be different than zero (i.e. at least one variable is included), making the range of the regularisation hyper-parameters easier to define.

### 2.3 Learning and validation framework

The proposed framework is divided into three main parts: hyper-parameter optimisation, statistical evaluation, and matrix deflation. These will be addressed in Sections 2.3.1, 2.3.2, 2.3.3, respectively.

#### 2.3.1 Hyper-parameter optimisation

Several studies have used *k*-fold cross-validation to select the optimal model hyper-parameters using the correlation between the projections of the data onto the weight vectors as a metric (Parkhomenko et al., 2009). Since the number of samples available in neuroimaging datasets is usually small, the natural tendency would be to use more folds with a lower number of samples per fold, which increases the variance of the cross-validation results. To overcome this issue, the proposed framework uses an approach based on random subsampling of the data.

**Fig. 1:**
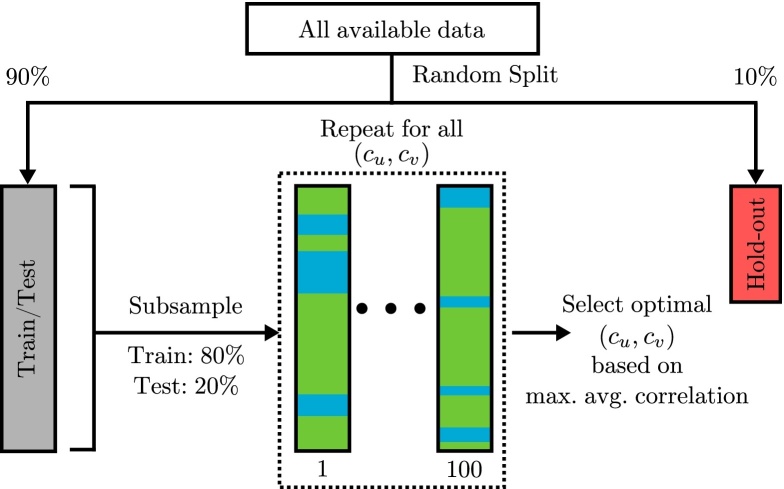
Hyper-parameter optimisation framework.

The proposed framework starts by removing 10% of the data randomly and keeping it as a hold-out dataset (Fig. 1), which will be used later for the statistical evaluation (Section 2.3.2). Then, the train/test dataset is randomly split 100 times into a training set (80% of the data) and a testing set (20% of the data). For each split, the model is trained on the training set, the testing data are projected onto the resulting weight vector pair, and the correlation between the projections of the two views is computed:

(5) $$\rho_{k}=|\mathrm{Corr}({\text{X}}_{k}{\text{u}}_{\left(-k\right)},{\text{Y}}_{k}{\text{v}}_{\left(-k\right)})|$$

where **X**_*k*_ and **Y**_*k*_ denote the testing sets; and **u**_(−*k*)_ and **v**_(−*k*)_ are the weight vectors computed using the training data.

The average correlation of *K* splits (where *K* = 100) for a specific hyper-parameter combination $\left(c_{u},c_{v}\right)$ is then computed using the arithmetic mean: ${\bar{\rho }}_{c_{u},c_{v}}=\frac{1}{K}\sum_{k=1}^{K}\rho_{k}$. This procedure is repeated for several hyper-parameter combinations spanning the full hyper-parameter range, and the combination with the highest average correlation is selected, i.e. a grid-search is performed. The selected hyper-parameter combination will then be used to train the models in the statistical evaluation step (Section 2.3.2).

By doing this random subsampling procedure, we are increasing both the number of correlation values (*ρ*_*k*_) used to compute the average correlation (${\bar{\rho }}_{c_{u},c_{v}}$), and the size of the testing datasets, which should make the estimation of the average correlation per hyper-parameter combination (${\bar{\rho }}_{c_{u},c_{v}}$) more stable. Please note that the same random splits are performed for each hyper-parameter combination. Also, the grid-search is performed using 40 equidistant points in $1⩽c_{u}⩽\sqrt{p}$ and $1⩽c_{v}⩽\sqrt{q}$, which makes a total of 1600 hyper-parameter combinations. The plots showing the average absolute correlation for different hyper-parameter values (hyper-parameter space) are provided in the supplementary material of this paper.

#### 2.3.2 Statistical evaluation

When testing the statistical significance of an associative effect using a permutation test with a nested cross-validation framework, one has to re-train the model for every permutation, including the hyper-parameter optimisation step. Unfortunately, when dealing with very high-dimensional data, such as whole-brain images, this can be computationally prohibitive. In order to assess the statistical significance of the weight vector pairs without performing a hyper-parameter optimisation for each permutation, we propose an approach which uses hold-out datasets {X^*^, Y^*^}.

**Fig. 2:**
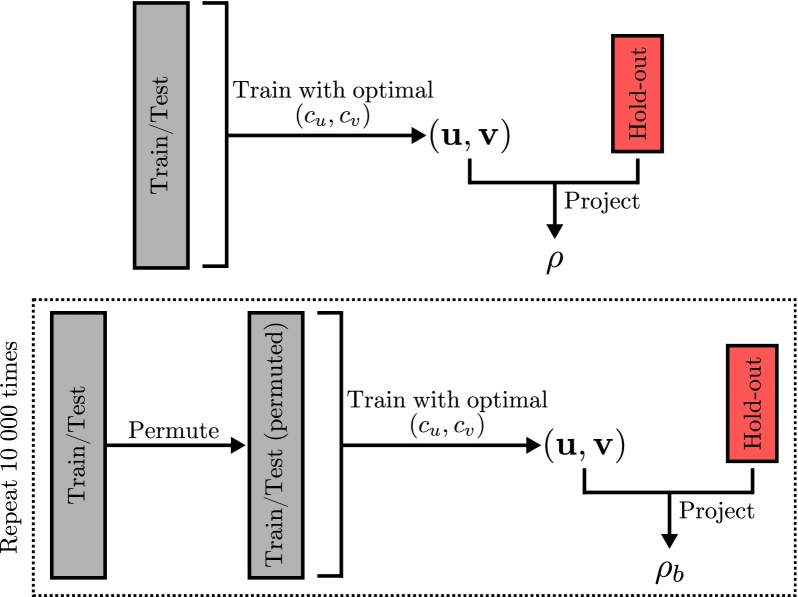
Permutation framework.

The statistical evaluation step is summarised in Fig. 2, which starts by training a model with all the train/test data using the optimal hyper-parameters selected in the previous step (Section 2.3.1), the hold-out data are projected onto these vectors and the absolute correlation between the projections is computed:

(6) $$\rho =|\mathrm{Corr}({\text{X}}^{*}\text{u},{\text{Y}}^{*}\text{v})|$$

where **u** and **v** are the weight vectors obtained by training the model with the train/test dataset.

During the permutation, the order of the samples in one of the views is permuted while leaving the other view untouched, this will destroy the relationship between the two views. The model is then trained again with the permuted data and the absolute correlation between the projections is computed:

(7) $$\rho_{b}=|\mathrm{Corr}({\text{X}}^{*}{\text{u}}_{b},{\text{Y}}^{*}{\text{v}}_{b})|$$

where **u**_*b*_ and **v**_*b*_ are the weight vectors obtained by training the model with the permuted train/test dataset for permutation *b*. The process is then repeated *B* times (re-training the model with the permuted dataset, and projecting the hold-out data onto the computed weight vectors).

Finally, the statistical significance of the weight vector pair is tested using the following null-hypothesis, *H*_*s*_: “There is no relationship between the two views, therefore the correlation obtained with the original data is not different from the correlation obtained with the permuted data”. If the probability of obtaining a correlation as large (or larger) than the original one is very low, i.e. *p*-value is very low, then we can reject the null-hypothesis and conclude that the found associative effect is statistically significant.

The *p*-value is calculated in the following way:

(8) $$p=\frac{1+\sum_{b=1}^{B}1_{\rho_{b}⩾\rho }}{B+1}$$

where *B* is the number of permutations, *ρ* is the correlation obtained when training the models using the non-permuted data, and *ρ*_*b*_ is the correlation obtained when training the models using the permuted dataset during permutation *b*. The addition of 1 in the nominator and denominator of Eq. (8) is equivalent to including *ρ* in the permutations. This will guarantee that *p* > 0 and that a minimum number of permutations will have to be performed in order to obtain a low enough *p*-value.

Due to the small sample sizes usually associated with neuroimaging datasets, the *p*-value may be estimated using a small hold-out dataset, which can lead to high variance in the results, depending on how the data were split. In order to make the model estimation more robust, the proposed framework uses several hold-out datasets. After the *p*-value is obtained, the process goes back to the beginning (Section 2.3.1) and is repeated 9 times, which means that in the end 10 *p*-values are obtained: one for each random split of the hold-out dataset. A criteria is then necessary to determine if there are any statistically significant effects in the data that are being fitted by SPLS, this can be done by using the concept of the *omnibus hypothesis*. The omnibus hypothesis was previously proposed for mass-univariate statistical tests, where a statistical test is performed on each voxel *j* in region *R*, in order to test a null-hypothesis *H*_*j*_. In this case, the combined hypothesis *H*_*R*_ over all the voxels is the following: “All the hypothesis *H*_*j*_ are true”. This is known as the omnibus hypothesis and, as one can see, will be rejected if any of the *H*_*j*_ hypothesis is rejected (Holmes, 1994, Nichols and Holmes, 2001). In the present work, we propose the use of this concept to evaluate groups of random splits of the data. In this case, the omnibus hypothesis *H*_omni_ is: “All the null-hypothesis *H*_*s*_ are true”. In other words, if any of the 10 *p*-values (obtained using the 10 random splits of the data) is statistically significant, then, the omnibus hypothesis will be rejected. All the *p*-values should be corrected for multiple comparisons by performing a Bonferroni correction, i.e. in order to have a family-wise error rate of 0.05: *α* = 0.05/10 = 0.005. Therefore, the omnibus hypothesis will be rejected if any of the 10 splits has *p* ⩽ 0.005.

Finally the statistically significant weight vector pair with the lowest *p* will be selected to be used for matrix deflation (Section 2.3.3). In case several weight vector pairs have the same *p*-value, the one with the highest hold-out correlation (Eq. (6)) is selected.

#### 2.3.3 Matrix deflation

If the omnibus hypothesis is rejected (Section 2.3.2), then the effect found by SPLS is statistically significant and needs to be removed from the data, in order to look for potential additional effects. This is done by matrix deflation, removing the effect described by the weight vector pair *h* before computing the next pair (*h* + 1). Witten et al. described the following deflation method (Witten et al., 2009):

(9) $$\begin{array}{c} d_{h}={\text{u}}_{h}^{⊤}{\text{C}}_{h}{\text{v}}_{h}\\ {\text{C}}_{h+1}\leftarrow {\text{C}}_{h}-d_{h}{\text{u}}_{h}^{⊤}{\text{v}}_{h} \end{array}$$

This type of deflation is known as Hotelling's deflation, which is used in several algorithms, including Principal Component Analysis (PCA) (Mackey, 2008). However, as noted by Mackey (2008), PCA extracts the leading eigenvectors of a variance matrix. When a sparse version of PCA is used (SPCA), this extracts *pseudo-eigenvectors*, which do not have the same properties as regular eigenvectors. Thus, the orthogonality property of the Hotelling's deflation does not hold. When SPLS is used in neuroimaging with non-orthogonal weight vector pairs, it can lead to cases where these will express the same effect, instead of finding new effects after each deflation (Monteiro et al., 2014). In order to avoid this, the proposed framework uses a different deflation method, which tries to force the orthogonality of the weight vector pairs. This method removes the covariance explained by **u**_*h*_ and **v**_*h*_ from **X** and **Y**, by projecting the data matrices onto the space spanned by the corresponding weight vector, and subtracting this from the data:

(10) $$\begin{array}{c} {\text{X}}_{h+1}\leftarrow {\text{X}}_{h}({\text{I}}_{x}-{\text{u}}_{h}{\text{u}}_{h}^{⊤})={\text{X}}_{h}-({\text{X}}_{h}{\text{u}}_{h}){\text{u}}_{h}^{⊤}\\ {\text{Y}}_{h+1}\leftarrow {\text{Y}}_{h}({\text{I}}_{y}-{\text{v}}_{h}{\text{v}}_{h}^{⊤})={\text{Y}}_{h}-({\text{Y}}_{h}{\text{v}}_{h}){\text{v}}_{h}^{⊤} \end{array}$$

This method has been previously described for SPCA as “projection deflation” (Mackey, 2008). Another disadvantage of the Hotelling's deflation is that it is applied to the covariance matrix and not to the individual data matrices, which would make it incompatible with the current framework.

Finally, there is a deflation step which has been used in SPLS (Lê Cao et al., 2008, Lê Cao et al., 2009), but is inherited from PLS (Wegelin, 2000):

(11) $$\begin{array}{c} \xi_{h}={\text{X}}_{h}\frac{{\text{u}}_{h}}{{\text{u}}_{h}^{⊤}{\text{u}}_{h}},\;\omega_{h}={\text{Y}}_{h}\frac{{\text{v}}_{h}}{{\text{v}}_{h}^{⊤}{\text{v}}_{h}}\\ {\text{a}}_{h}={\text{X}}_{h}^{⊤}\frac{\xi_{h}}{\xi_{h}^{⊤}\xi_{h}},\;{\text{b}}_{h}={\text{Y}}_{h}^{⊤}\frac{\omega_{h}}{\omega_{h}^{⊤}\omega_{h}}\\ {\text{X}}_{h+1}\leftarrow {\text{X}}_{h}-\xi_{h}{\text{a}}_{h}^{⊤}\\ {\text{Y}}_{h+1}\leftarrow {\text{Y}}_{h}-\omega_{h}{\text{b}}_{h}^{⊤} \end{array}$$

This deflation approach was compared with the projection deflation approach used in the proposed framework.

The SPLS results were also compared with the results acquired by applying the same framework with PLS. Please note that in this case there are no hyper-parameters to optimise, thus, the step described in Section 2.3.1 is not performed.

### 2.4 Projection onto the SPLS latent space

The data can be projected onto the statistically significant weight vectors from both image and clinical views: ***ξ***_*h*_ = **Xu**_*h*_ , ***ω***_*h*_ = **Yv**_*h*_. As previously mentioned, these weight vectors represent the SPLS latent space. The projection of the data onto this space may bring insights about their structure, which can potentially be used for patient stratification.

### 2.5 Dataset

The data used in the preparation of this article were obtained from the Alzheimer's Disease Neuroimaging Initiative (ADNI) database (adni.loni.usc.edu). The ADNI was launched in 2003 as a public-private partnership, led by Principal Investigator Michael W. Weiner, MD. The primary goal of ADNI has been to test whether serial Magnetic Resonance Imaging (MRI), Positron Emission Tomography (PET), other biological markers, and clinical and neuropsychological assessment can be combined to measure the progression of mild cognitive impairment (MCI) and early Alzheimer's disease (AD). For up-to-date information, see www.adni-info.org.

SPLS was applied to investigate the association between the grey matter maps and the individual scores of the questions/tasks of the MMSE, which is a quite widely used exam that is performed on patients with dementia (Folstein et al., 1975). The dataset consisted of a subset of 592 unique subjects from the ADNI: 309 males (average age 74.68 ± 7.36) and 283 females (average age 72.18 ± 7.50). These subjects were clinically labeled as being either healthy, suffering from MCI, or suffering from AD. The T1 weighted MRI scans were segmented into grey matter probability maps using SPM12, normalised using DARTEL, converted to MNI space with voxel size of 2 mm × 2 mm × 2 mm and smoothed with a Gaussian filter with 2 mm FWHM. A mask was then generated, this selected voxels which had an average probability of being grey matter equal or higher than 10% for the whole dataset. This resulted in 168 130 voxels per subject being used.

Each question/task of the MMSE was coded in the following way: the subjects were given a score of 1 if the answer was correct, or the task was performed correctly; and a score of 2 if the answer was wrong, or the task was not performed correctly. The exam is conducted by a clinician and is divided into five categories, each containing different questions/tasks, which test five different cognitive domains (Folstein et al., 1975):

• Orientation (questions 1 to 10) — These are related with temporal and spatial orientation.
• Registration (questions 11 to 13) — The clinician names three objects and asks the patient to repeat all three. There is an extra question (13.a) in which the clinician writes down the number of trials that the subject had to take.
• Attention and Calculation (questions 14 to 18) — The subject is asked to spell the word “world” backwards (i.e. “D”, “L”, “R”, “O”, “W”). A score is attributed for each letter, and the subject is only given a good score if the letter is in the correct order.
• Recall (questions 19 to 21) — The subject is asked to name the three objects named before (questions 11 to 13).
• Language (questions 22 to 30) — These questions/tasks involve recognising and naming objects (e.g. naming a watch and a pencil), repeating a sentence, understanding verbal commands (e.g. “take a paper with the right hand”, “fold it in half”, “put it on the floor”), reading, writing, and drawing.

For a detailed list of the questions/tasks, please refer to Appendix A. All the features in both views (image and clinical) were mean-centered and normalised to have standard deviation equal to 1.

## 3 Results

### 3.1 Statistical significance testing

Table 1 shows the *p*-values obtained by using PLS with the proposed framework, as we can see, the omnibus hypothesis *H*_omni_ (Section 2.3.2) could not be rejected, i.e. *p* > 0.005 for all the splits. Although the proposed framework would stop as soon as a statistically significant weight vector pair could not be found, i.e. in the first associative effect for the considered dataset, the algorithm was run until 3 associative effects were found, in order to assess how the different deflation methods behave with PLS and SPLS.

The *p*-values obtained with SPLS can be seen in Table 2. In this case, *H*_omni_ was rejected twice: for the first and second associative effects using projection deflation. No statistically significant results were obtained when using a PLS deflation.

### 3.2 Generalisability of the weight vectors

**Fig. 3:**
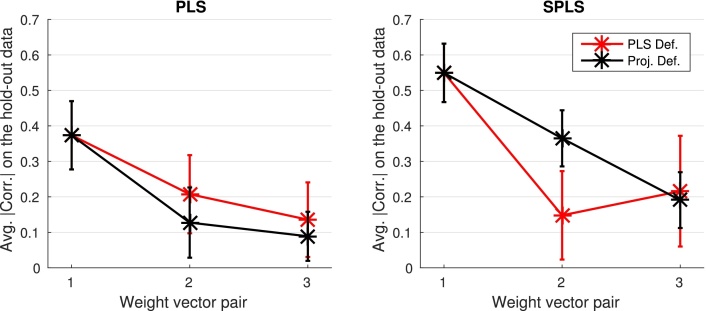
Average absolute correlation on the hold-out datasets.

Fig. 3 shows the average absolute correlation on the 10 hold-out datasets obtained with both PLS and SPLS, using the two types of deflation. The average absolute correlation on the hold-out datasets exhibits a consistent downward trend, and seems to be higher when PLS deflation is applied with PLS. However, when SPLS is used, projection deflation seems to perform better, exhibiting higher average correlation values on the hold-out datasets, and having smaller standard deviation (which is reflected by the smaller error bars).

### 3.3 Weight vectors or associative effects

#### 3.3.1 PLS

Since PLS was not able to reject the omnibus hypothesis, no weight vectors are presented in this section. For comparative purposes, the average of the weight vectors for the first effect can be seen in Appendix B.1.

#### 3.3.2 SPLS

**Fig. 4:**
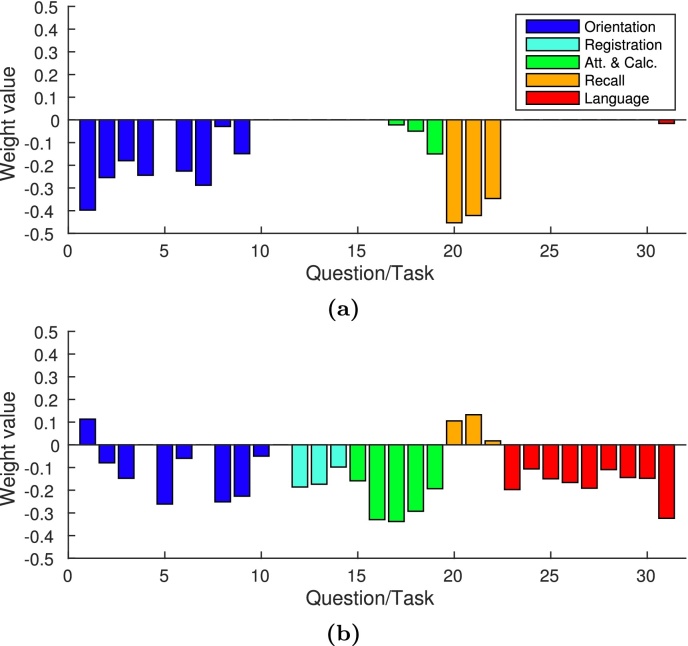
(a) First clinical weight vector; (b) Second clinical weight vectors. The sign of the second weight vector was inverted for visualisation only (in order to be consistent with the first weight vector pair).

**Fig. 5:**
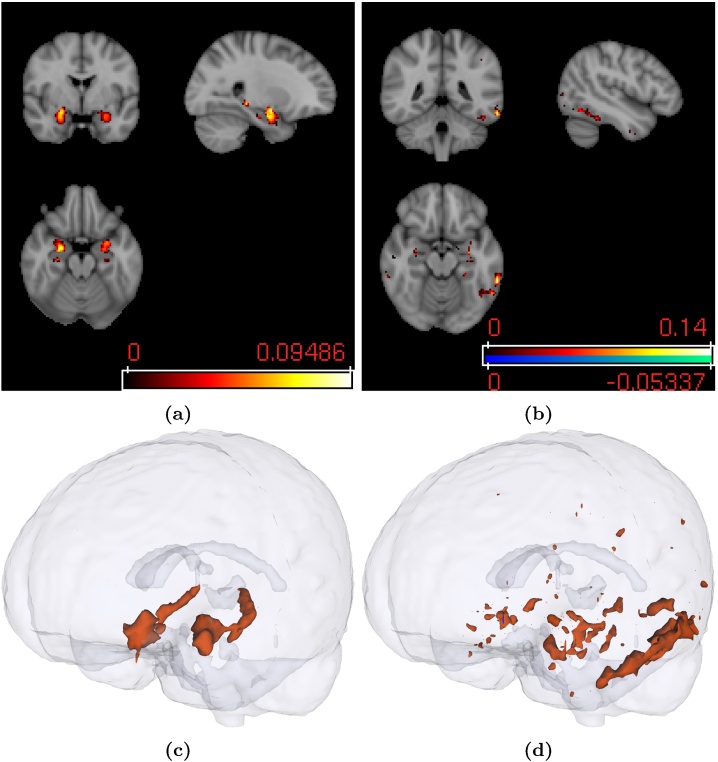
(a) First image weight vector; (b) Second image weight vector; (c) 3D visualisation of the features selected for the first image weight vector; (d) 3D visualisation of the features selected for the second image weight vector. Red regions denote positive weights and blue regions denote negative weights (very small region on the second weight vector). The sign of the second weight vector was inverted for visualisation purposes only (in order to be consistent with the first weight vector pair). (For interpretation of the references to colour in this figure legend, the reader is referred to the web version of this article.)

Unlike PLS, SPLS found statistically significant sparse weight vectors, representing associative effects between clinical (Fig. 4) and image views (Fig. 5). This section will only present the statistically significant weight vectors, which were obtained using projection deflation. For comparative purposes, the averages of the weight vectors for the second effect using PLS deflation and projection deflation are presented in Appendix B.2.

##### 3.3.2.1 First associative effect

As previously mentioned, each weight vector pair represents a multivariate associative effect between the two views (brain voxels and clinical variables), i.e. the clinical weight vector will show a subset of clinical variables associated with a subset of brain voxels displayed in the image weight vector. Fig. 4(a) shows the first clinical weight vector. It is possible to see that only 15 out of 31 clinical variables were selected. These belonged mainly to the “Orientation”, “Attention and Calculation”, and “Recall” domains. One variable was selected in the “Language” domain. The weight vector corresponding to the first image weight vector can be seen in Fig. 5(a). As we can see, the weight map is very sparse and the regions found have been previously associated with memory (e.g. hippocampus and amygdala) (Jack et al., 2000).

Using the Automated Anatomical Labeling (AAL) atlas (Tzourio-Mazoyer et al., 2002), it is possible to summarise the image weight vectors by ranking the regions of the atlas by their average absolute weight value. The average was used to take into account the different atlas region sizes, i.e. the larger the fraction of voxels equal to zero in a region is, the lower the average absolute weight in that region will be. Table 3 shows the top 10 regions for the first image weight vector. For the complete list of regions, please refer to the supplementary material of the paper.

##### 3.3.2.2 Second associative effect

The second clinical weight vector was not as sparse as the previous one (Fig. 4(b)): 28 out of 31 variables were selected. The magnitudes of the weights for the “Recall” domain are substantially smaller than on the previous weight vector pair, while the absolute values of the weights on the “Registration”, “Attention and Calculation”, and “Language” domains are greater. The voxels found by the second image weight vector (Fig. 5(b) and (d)) were less localised than the ones in the first image weight vector, these were present mostly in the temporal lobes, hippocampus, and amygdala. The second associative effect seems to capture an association between all domains of the MMSE score and mainly temporal regions in the left brain hemisphere.

The top 10 regions for the second image weight vector can be seen in Table 4. For the complete list of regions, please refer to the supplementary material of the paper.

Please note that most voxels in Fig. 5(a) and (b) have positive weights, while most signs of the corresponding clinical weight vector have negative weights (Fig. 4(a) and (b)). This means that both effects follow the same tendency: high grey matter density (high image weights) are associated with generally low values in the clinical questions/tasks (i.e. the task was performed correctly, Section 2.5), and *vice versa*.

### 3.4 Projection onto the SPLS latent space

All the available data were projected onto the weight vector pairs computed using SPLS, in order to bring insights about structure in the data, and to potentially stratify patients (Section 2.4). Since PLS was not able to find statistically significant weight vector pairs, the projections for this method will not be presented.

**Fig. 6:**
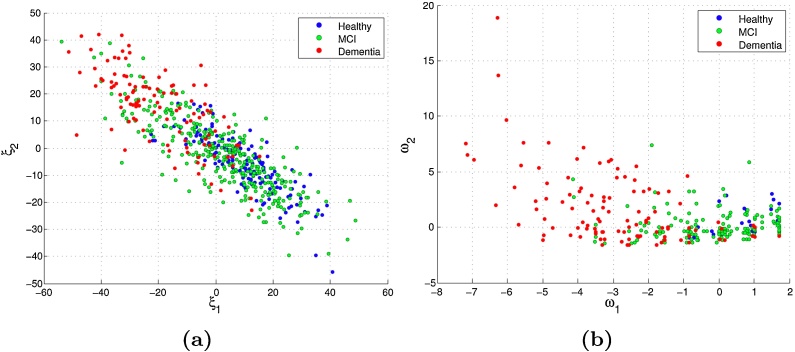
(a) Projection of the image data onto the image weights; (b) Projection of the clinical data onto the clinical weights.

Fig. 6(a) shows the projection of the data onto both SPLS image weight vectors, while Fig. 6(b) shows the projection of the data onto both SPLS clinical weight vectors. Each point represents the projection of one subject's data onto the subspace defined by the weight vector pair, where its color is given based on the clinical diagnosis. The horizontal axes (***ξ***_1_ and ***ω***_1_) correspond to the projections onto the first weight vector, and the vertical axes (***ξ***_2_ and ***ω***_2_) correspond to the projections onto the second vector.

As we can see, there are no defined clusters, however, there seems to be a continuous distribution of subjects from lower to higher degrees of neurodegeneration.

## 4 Discussion

Our results show that the proposed SPLS framework was able to detect two statistically significant associative effects between grey matter maps and individual questions/tasks of the MMSE score when using sparsity constraints on both views. To the best of our knowledge, this has not been previously shown. These results are particularly interesting as the information encoded on the individual question/task level is very noisy, however, it also expresses more subtle effects in the data when compared with a summarised final exam score. The first effect captured an association mainly between the “Orientation”, “Attention and Calculation” and the “Recall” domains on the clinical view, and brain regions such as the amygdala and hippocampus. While the second effect captured an association between most clinical variables, and regions mainly on the left brain hemisphere, including temporal regions. These results were achieved by imposing sparsity in both views, and without using any *a priori* assumption regarding data structure, which might be useful when it is not possible to have one. Moreover, the projection of the subjects onto the latent SPLS space showed a consistent distribution of the subjects from lower to higher degrees of neurodegeneration.

Projection deflation has shown to provide more reliable weight vectors when compared with the commonly used PLS deflation method. When comparing the different deflation approaches, the results showed that only by using projection deflation it was possible to find a second statistically significant associative effect with SPLS. Moreover, projection deflation provided a higher average correlation on the hold-out datasets, i.e. the model generalises better for unseen data.

The proposed framework was also tested with PLS. Our results showed that SPLS performed better than PLS, not only by being able to find statistically significant associative effects, but also by improving the interpretability of the weight vector pairs due to their sparsity, and by generalising better for unseen data (which can be demonstrated by an increase in average correlation obtained in hold-out datasets).

### 4.1 Multiple hold-out framework

In the present study, we proposed a SPLS framework which uses multiple random splits for the hold-out dataset. By performing a significance test on each random split, the framework checks how reliable the weight vector computation is to data perturbations, making it more robust than approaches based on a single split.

The estimation of the sparsity levels for both views without *a priori* assumptions allows for greater flexibility when trying to find the best model to describe a particular associative effect in the data. Moreover, these levels are not fixed for every weight vector pair, which means that each associative effect will be described by the right level of sparsity in each view, i.e. the proposed approach will find the necessary number of voxels and clinical variables to describe each associative effect.

One of the main advantages of the proposed framework when compared with a more widespread nested cross-validation approach is its computational speed. Nested cross-validation consists in performing a two level cross-validation, where, for each training fold, an inner cross-validation procedure is performed for every hyper-parameter combination, in order to select the optimal hyper-parameter combination to be applied in the outer fold. This is a quite computationally intensive procedure, since hyper-parameter selection will have to be repeated for each permutation during the statistical evaluation. Even if nested cross-validation were to be applied to this problem with a small number of folds (5 inner folds and 5 outer folds) and permutations (1000), it would require 40 045 005 SPLS computations, whereas the proposed framework with 100 subsamples (which should provide a more stable hyper-parameter selection) and 10 000 permutations, computed SPLS 1 700 010 times, which corresponds to approximately 4% of the number of computations that would have been necessary with a nested cross-validation framework. For the details of how these values are calculated, please refer to Appendix C.

Witten et al. proposed a hyper-parameter optimisation procedure based on permutations where, for each hyper-parameter combination, the *p*-value of the correlation using all the data was computed and the combination with the lowest *p* was selected (Witten and Tibshirani, 2009). This method will choose the hyper-parameters for which the distance between the true correlation and the null distribution of the correlations is the largest, however, this might not be the same hyper-parameters that maximise the correlation of the projections using testing data, which is what the proposed subsampling approach will try to achieve (Section 2.3.1). Moreover, the permutation based method may require a very large amount of permutations in order to enable correction for multiple comparisons.

### 4.2 Previous SPLS/SCCA applications to structural MRI and clinical scores

To our knowledge, using SPLS to study the effects in the brain by imposing sparsity on both voxels and individual questions/tasks of a cognitive exam has not been done before.

The investigation of multivariate associations between gray matter probability maps and clinical scores at the question/task level allows for greater flexibility, making the model unconstricted by pre-defined brain regions or sub-scales of clinical test scores. This may lead to the discovery of new associations that were not previously described in the literature.

Avants et al. have previously performed an analysis using structural brain images and the results of a clinical exam (Avants et al., 2014). However, the authors analysed each one of the domains separately without imposing sparsity on the clinical view (sparsity was only applied on the image view), whereas this experiment takes all questions from the MMSE at the same time with sparsity constraints on both views. This allows the model to exclude clinical variables which are not relevant when explaining the covariance associated with each effect. Moreover, Avants et al. also restricted their study to the first weight vector pair and used a different clinical exam (PBAC).

### 4.3 Statistical significance testing

SPLS with projection deflation was able to find two statistically significant associative effects in the data, while PLS was not able to find any. This result may be related to the fact that SPLS provided a higher average hold-out correlation (Fig. 3). The sparsity constraints used by SPLS allow the method to exclude noisy features, which will result in a model with a better ability to generalise for unseen data.

### 4.4 Comparison between deflation approaches

Projection deflation performed much better when applied to SPLS than the commonly used PLS deflation, being able to provide higher values of average correlation on the hold-out dataset (Fig. 3).

The role of the deflation step is to remove the associative effect expressed in the current weight vector pair from the data matrices. Both PLS and SPLS capture the strongest effect in the data with the first weight vector pair, after the covariance explained by this vector pair is removed, the signal-to-noise ratio in the data will decrease. This property allows the effects to be ranked, since each weight vector pair will explain more covariance in the data than the following ones. Our results suggest that using deflation strategies inherited from non-sparse methods might not be the best approach when dealing with sparse methods, these algorithms should have their own appropriate deflation steps.

#### 4.4.1 Multivariate associative effects

The first SPLS clinical weight vector is particularly interesting (Fig. 4(a)). The domains with the most prominent contribution to the weight vector were consistent with what it would be expected from a population with AD patients. All the questions in the “Recall” domain were chosen, while none of the questions in the “Registration” domain were selected. This suggests that whether patients remember words that were recently presented to them explains more covariance in the data than whether they repeat the words when they are first presented (which is reflected by the “Registration” domain). This was expected, since the inability to remember new information is one of the earliest and most prominent symptoms of AD (Galton et al., 2001). The regions associated with these variables, displayed by the image weight vector (Fig. 5(a), (c), and Table 3), have been previously described as being associated with dementia (Jack et al., 2000, Chan et al., 2001, Galton et al., 2001). Moreover, Table 3 and Fig. 5(c) show that there is symmetry in the regions selected (e.g. hippocampus, parahippocampal gyrus, and amygdala), which is consistent with the symmetric brain region atrophy that has been described in the literature (Chan et al., 2001, Galton et al., 2001).

Another interesting result is that only the last three variables (out of five) in the “Attention and Calculation” domain were selected, and with increasing absolute weight. During this part of the MMSE, the subject is trying to spell the word “world” backwards, each variable corresponds to whether the subject replied with the correct letter or not. The results seem to suggest that there might be some effect during this task that is dependent on the order of the letters.

Please note that unlike previous studies (Avants et al., 2014, Lin et al., 2014), no *a priori* assumptions regarding the structure of the data are used. The exclusion of virtually two entire domains (“Registration” and “Language”) was purely data-driven. Also, no information regarding brain structure was provided to the algorithm, e.g. region information based on a brain atlas.

For the second weight vector pair, it is interesting to see that most of the top regions are in the left side of the brain. Previous studies comparing AD with Semantic Dementia (SD), found that SD is characterised by a greater atrophy of structures on the left side of the brain (compared with the corresponding structures on the right side), particularly, the temporal lobe, parahippocampal gyrus, and fusiform gyrus (Chan et al., 2001, Galton et al., 2001). Patients with SD exhibit impairment of semantic memory, e.g. patients have difficulties with word meaning (Chan et al., 2001). SPLS selected these regions when the questions/tasks from the “Language” domain were included in the model (Fig. 4(b)), which suggests that the second weight vector pair is picking up effects primarily related with semantics, while the first is picking up effects primarily related with memory.

### 4.5 Projection onto the SPLS latent space

The projection of the subjects’ data onto the SPLS weight vectors (Fig. 6) shows that, although there are no defined clusters, the subjects seem to form a continuous distribution from healthier subjects, to progressively worse cases of neurodegeneration. It is also interesting to note that on the projections of the subjects onto the two clinical weight vectors (Fig. 6(b)), the second clinical weight vector does not seem to separate subjects based on diagnosis as well as the first, which suggests that the effect detected is less directly associated with the clinical labels.

### 4.6 Limitations and future work

Despite the promising results that were obtained with this framework, one of its possible limitations is the fact that the statistical evaluation step, using 10 different splits of the data with a Bonferroni correction for multiple comparisons, is rather strict, which might lead to some false negative results. Therefore, the framework may require large amounts of data in order to detect statistically significant associative effects, which may not be easily available. Nevertheless, the proposed framework should provide more reliable results, by evaluating multiple splits of the data.

In future work, it would be interesting to apply this framework to data in which clinical categories have shown to have limitations, such as, psychiatric data (Insel et al., 2010). In these cases, the use of SPLS to find associative effects between brain images and individual exam questions/tasks may provide valuable information that could help with patient stratification.

## 5 Conclusions

Our results represent a proof of concept that the proposed exploratory SPLS framework is able to find meaningful multivariate associations between the individual items from the MMSE score and a subset of relevant brain voxels from grey matter maps. The projection deflation method has shown to provide statistically significant results in the second weight vector pair (which was not observed when using the classic PLS deflation method), by trying to enforce orthogonality between the sparse weight vector pairs. In addition, by fitting the model to different splits of the data, we were able to obtain robust models and significance tests for SPLS. Finally, the different projections of the subjects onto the SPLS latent space showed that no defined clusters could be found, however, the subjects seemed to form a continuous distribution from healthier subjects, to progressively worse cases of neurodegeneration.

## Figures and Tables

**Table A1:** MMSE questions/tasks.

Domain	Question/task
Orientation	1. What is today's date?
2. What year is it?
3. What month is it?
4. What day of the week is today?
5. What season is it?
6. What is the name of this hospital?
7. What floor are we on?
8. What town or city are we in?
9. What county (district) are we in?
10. What state are we in?
Registration	11. Name object (ball)
12. Name object (flag)
13. Name object (tree)
13a. Number of trials
Att. & calc.	14. D
15. L
16. R
17. O
18. W
Recall	19. Recall Ball
20. Recall Flag
21. Recall Tree
Language	22. Show a wrist watch and ask “What is this?”
23. Show a pencil and ask “What is this?”
24. Repeat a sentence
25. Takes paper in right hand
26. Folds paper in half
27. Puts paper on floor
28. Read and obey a command (“Close your eyes”)
29. Write a sentence
30. Copy design

**Fig. A1:**
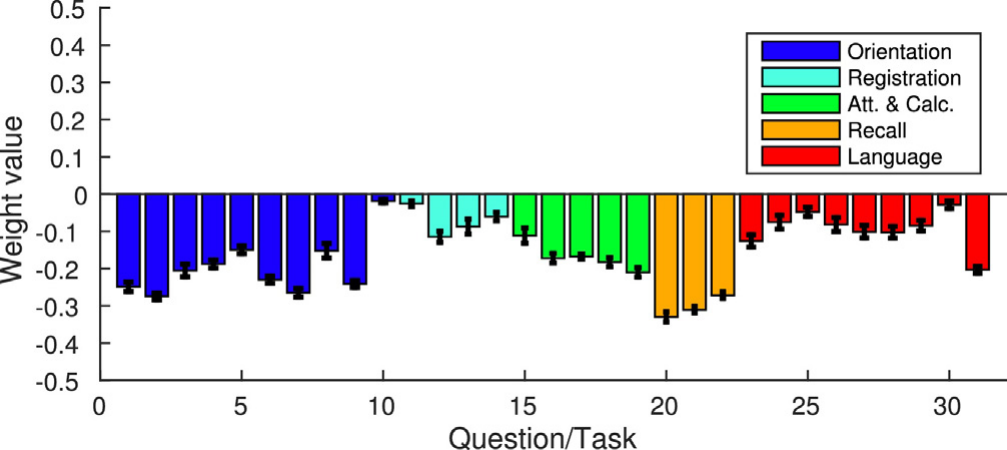
Mean of clinical weight vector using PLS.

**Fig. A2:**
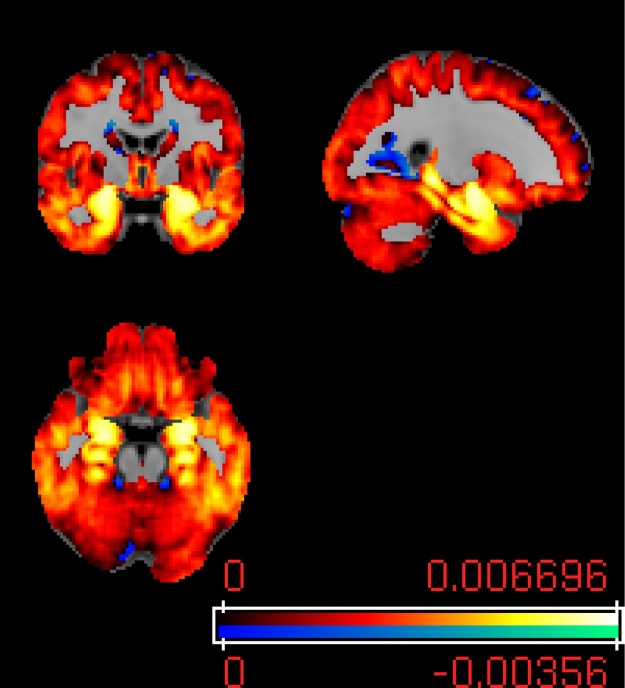
Mean of image weight vectors using PLS.

**Fig. A3:**
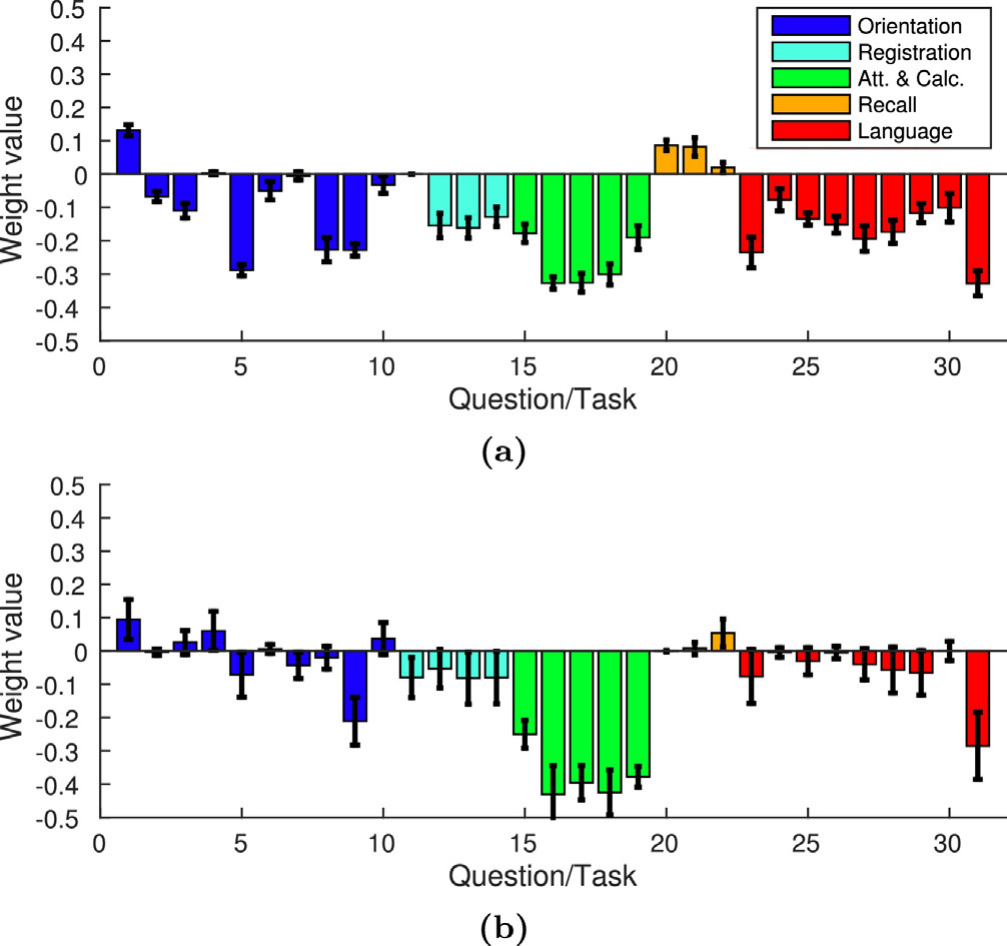
(a) Mean of second clinical weight vectors using SPLS with projection deflation; (b) Mean of second clinical weight vectors using SPLS with PLS deflation. The weights vectors in (b) were forced to have a consistent direction before averaging.

**Fig. A4:**
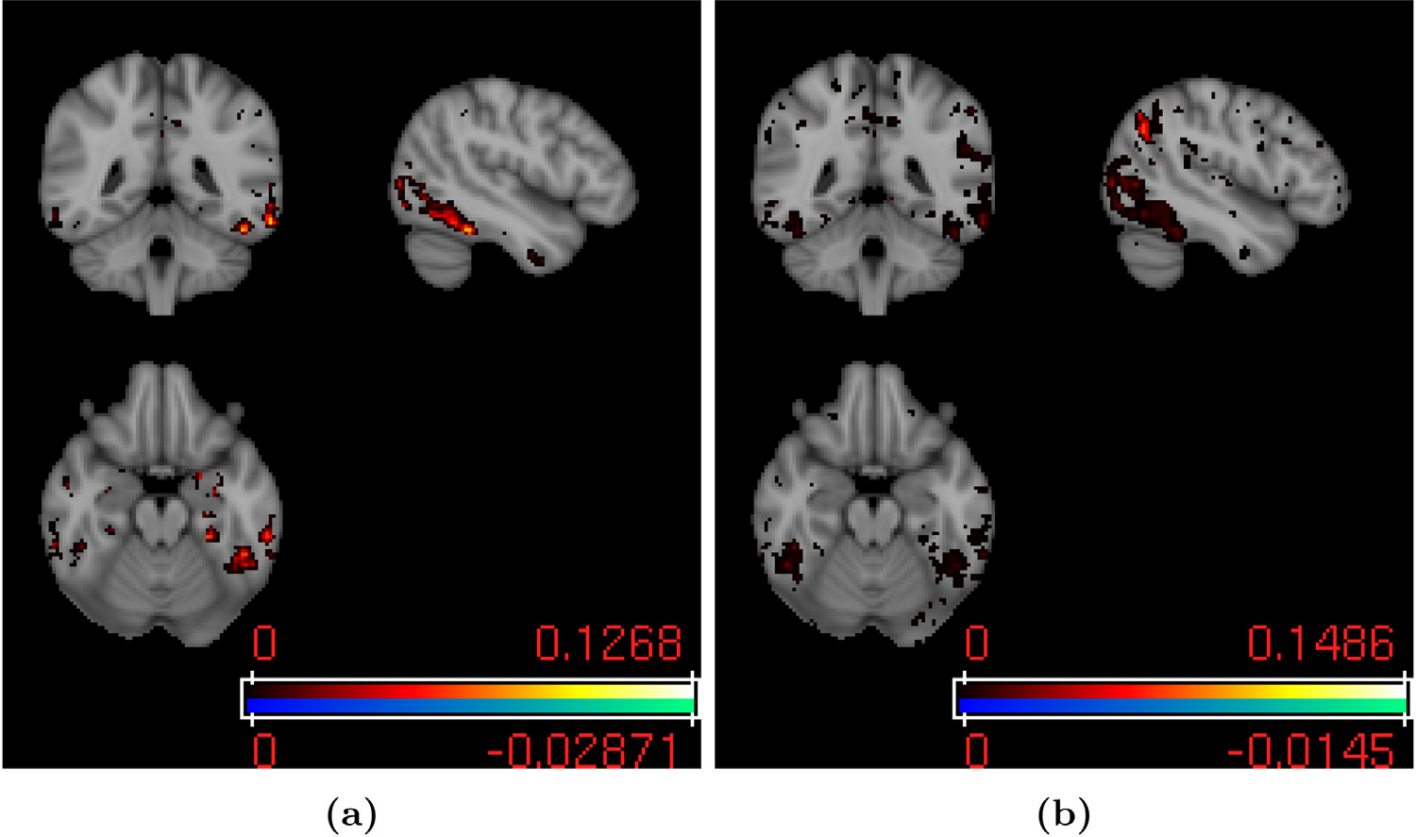
(a) Mean of second image weight vectors using SPLS with projection deflation; (b) Mean of second image weight vectors using SPLS with PLS deflation.

**Table 1:** PLS *p*-values computed with 10 000 permutations. All *p*-values are rounded to 4 decimal places.

	PLS (**u**, **v**) pair				
Split		PLS deflation		Proj. deflation	
	1	2	3	2	3
1	0.0690	0.0193	0.8619	0.4635	0.9853
2	0.2825	0.0655	0.0422	0.0323	0.3175
3	0.0120	0.2718	0.0173	0.4599	0.0609
4	0.0902	0.4255	0.4968	0.0742	0.4836
5	0.0924	0.9607	0.9855	0.9152	0.2106
6	0.0844	0.3984	0.1593	0.3412	0.5270
7	0.0866	0.1860	0.7745	0.9767	0.8342
8	0.0894	0.0479	0.1417	0.3052	0.6869
9	0.1233	0.1396	0.3932	0.3170	0.5775
10	0.0224	0.0289	0.1805	0.9831	0.7565
Rej. *H*_omni_	No	No	No	No	No

**Table 2:** SPLS *p*-values computed with 10 000 permutations (statistically significant results are shown in bold). All *p*-values are rounded to 4 decimal places.

	SPLS (**u**, **v**) pair				
Split		PLS deflation		Proj. deflation	
	1	2	3	2	3
1	**0.0007**	0.2476	0.3754	0.0376	0.0583
2	0.0068	0.2365	0.3585	**0.0041**	0.1769
3	**0.0002**	0.6051	0.0460	0.5298	0.5029
4	**0.0002**	0.9637	0.2013	0.0509	0.2841
5	**0.0001**	0.5711	0.9273	**0.0012**	0.3978
6	**0.0005**	0.6613	0.1107	0.0782	0.3267
7	**0.0001**	0.6073	0.3526	0.0256	0.0066
8	**0.0016**	0.9777	0.4515	0.0405	0.1126
9	**0.0004**	0.0713	0.4301	**0.0002**	0.0692
10	**0.0001**	0.1618	0.1817	0.2745	0.4399
Rej. *H*_omni_	**Yes**	No	No	**Yes**	No

**Table 3:** Top 10 atlas regions for the first image weight vector.

Atlas region	# voxels found
Amygdala_L	98
Amygdala_R	90
Hippocampus_R	175
Hippocampus_L	152
ParaHippocampal_R	92
ParaHippocampal_L	44
Lingual_L	9
Precuneus_L	2
Precuneus_R	1
Temporal_Pole_Sup_L	1

**Table 4:** Top 10 atlas regions for the second weight vector.

Atlas region	# voxels found
Amygdala_L	36
Temporal_Inf_L	292
Hippocampus_L	88
Amygdala_R	11
ParaHippocampal_L	53
Fusiform_L	78
Temporal_Inf_R	64
Hippocampus_R	22
Occipital_Inf_L	12
Temporal_Mid_L	76

## Appendix A Mini-Mental State Examination

Table A1 gives a brief description of the questions/tasks performed during the MMSE (Folstein et al., 1975).

## Appendix B Weight vectors or associative effects

### B.1 PLS

The average of the clinical weight vectors is presented in Fig. A1, and the corresponding image weight vector can be seen in Fig. A2. As expected, these are not sparse, all the available clinical variables and image voxels are included in the model. As one can see, the weights are higher in the hippocampus and amygdala regions. However, since these are weights and not *p*-values (as the ones obtained in an mass-univariate statistical test), they cannot be thresholded.

### B.2 SPLS with PLS deflation

This section shows the results obtained for the second weight vector pair when the common PLS deflation (Eq. (11)) was performed, instead of the projection deflation (Eq. (10)).

Fig. A3(b) shows the mean clinical weight vector obtained using a PLS deflation. The weight vectors are less reliable than the ones obtained with SPLS using projection deflation (Fig. A3(a)), which can be observed by the larger standard deviation bars. Also, the weight vectors in Fig. A3(b) had to be forced to have a consistent direction before averaging, i.e. the direction of the weight vectors flipped from one split to another, which was not observed when using projection deflation (the direction was consistent). If this was not done, the standard deviation bars for the PLS deflation would be even larger. Thus, it is interesting to see that the non-statistical significance of the weight vectors is consistent with their lack of reliability.

The mean second image weight vector computed using the projection deflation is shown in Fig. A4(a), this appears to be sparser than the one obtained when using SPLS with a PLS deflation (Fig. A4(b)).

## Appendix C Number of SPLS computations

In order to statistically evaluate one weight vector pair, the number of times SPLS has to be performed in a nested cross-validation is given by:

(C.1) $$N_{\mathrm{SPLS}}=(k_{o}(n_{p}k_{i})+k_{o})+B(k_{o}(n_{p}k_{i})+k_{o})$$

where *k*_*o*_ is the number of outer folds, *k*_*i*_ is the number of inner folds, *n*_*p*_ is the number of hyper-parameter combinations, and *B* is the number of permutations.

The proposed framework requires a number of SPLS computations given by:

(C.2) $$N_{\mathrm{SPLS}}=S(Kn_{p}+1)+\mathrm{SB}$$

where *S* is the number of hold-out splits, and *K* is the number of subsample splits.

## References

[bib0005] Avants B.B., Cook P.A., Ungar L., Gee J.C., Grossman M. (2010). Dementia induces correlated reductions in white matter integrity and cortical thickness: a multivariate neuroimaging study with sparse canonical correlation analysis. Neuroimage.

[bib0010] Avants B.B., Libon D.J., Rascovsky K., Boller A., McMillan C.T., Massimo L., Coslett H.B., Chatterjee A., Gross R.G., Grossman M. (2014). Sparse canonical correlation analysis relates network-level atrophy to multivariate cognitive measures in a neurodegenerative population. Neuroimage.

[bib0015] Chan D., Fox N., Scahill R., Crum W., Whitwell J., Leschziner G., Rossor A., Stevens J., Cipolotti L., Rossor M. (2001). Patterns of temporal lobe atrophy in semantic dementia and Alzheimer's disease. Ann. Neurol..

[bib0020] Della-Maggiore V., Sekuler A.B., Grady C.L., Bennett P.J., Sekuler R., McIntosh A.R. (2000). Corticolimbic interactions associated with performance on a short-term memory task are modified by age. J. Neurosci..

[bib0025] Ecker C., Marquand A., Mourão-Miranda J., Johnston P., Daly E.M., Brammer M.J., Maltezos S., Murphy C.M., Robertson D., Williams S.C. (2010). Describing the brain in autism in five dimensions-magnetic resonance imaging-assisted diagnosis of autism spectrum disorder using a multiparameter classification approach. J. Neurosci..

[bib0030] Folstein M.F., Folstein S.E., McHugh P.R. (1975). Mini-mental state. A practical method for grading the cognitive state of patients for the clinician. J. Psychiatr. Res..

[bib0035] Galton C.J., Patterson K., Graham K., Lambon-Ralph M.A., Williams G., Antoun N., Sahakian B.J., Hodges J.R. (2001). Differing patterns of temporal atrophy in Alzheimer's disease and semantic dementia. Neurology.

[bib0040] Giessing C., Fink G.R., Rösler F., Thiel C.M. (2007). fMRI data predict individual differences of behavioral effects of nicotine: a partial least square analysis. J. Cogn. Neurosci..

[bib0045] Holmes A.P. (1994). Statistical Issues in Functional Brain Mapping.

[bib0050] Hotelling H. (1936). Relations between two sets of variates. Biometrika.

[bib0055] Insel T., Cuthbert B., Garvey M., Heinssen R., Pine D.S., Quinn K., Sanislow C., Wang P. (2010). Research domain criteria (RDoC): toward a new classification framework for research on mental disorders. Am. J. Psychiatry.

[bib0060] Jack C., Petersen R., Xu Y., O’brien P., Smith G., Ivnik R., Boeve B., Tangalos E., Kokmen E. (2000). Rates of hippocampal atrophy correlate with change in clinical status in aging and AD. Neurology.

[bib0065] Keightley M.L., Winocur G., Graham S.J., Mayberg H.S., Hevenor S.J., Grady C.L. (2003). An fMRI study investigating cognitive modulation of brain regions associated with emotional processing of visual stimuli. Neuropsychologia.

[bib0070] Keightley M.L., Seminowicz D.A., Bagby R.M., Costa P.T., Fossati P., Mayberg H.S. (2003). Personality influences limbic-cortical interactions during sad mood induction. Neuroimage.

[bib0075] Klöppel S., Stonnington C.M., Chu C., Draganski B., Scahill R.I., Rohrer J.D., Fox N.C., Jack C.R., Ashburner J., Frackowiak R.S. (2008). Automatic classification of MR scans in Alzheimer's disease. Brain.

[bib0080] Krishnan A., Williams L.J., McIntosh A.R., Abdi H. (2011). Partial least squares (PLS) methods for neuroimaging: a tutorial and review. Neuroimage.

[bib0085] Lê Cao K.-A., Rossouw D., Robert-Granié C., Besse P. (2008). A sparse PLS for variable selection when integrating omics data. Stat. Appl. Genet. Mol. Biol..

[bib0090] Lê Cao K.-A., Martin P.G.P., Robert-Granié C., Besse P. (2009). Sparse canonical methods for biological data integration: application to a cross-platform study. BMC Bioinf..

[bib0095] Labus J.S., Van Horn J.D., Gupta A., Alaverdyan M., Torgerson C., Ashe-McNalley C., Irimia A., Hong J.-Y., Naliboff B., Tillisch K., Mayer E.A. (2015). Multivariate morphological brain signatures predict chronic abdominal pain patients from healthy control subjects. Pain.

[bib0100] Le Floch E., Guillemot V., Frouin V., Pinel P., Lalanne C., Trinchera L., Tenenhaus A., Moreno A., Zilbovicius M., Bourgeron T., Dehaene S., Thirion B., Poline J.B., Duchesnay E. (2012). Significant correlation between a set of genetic polymorphisms and a functional brain network revealed by feature selection and sparse partial least squares. Neuroimage.

[bib0105] Lin D., Calhoun V.D., Wang Y.P. (2014). Correspondence between fMRI and SNP data by group sparse canonical correlation analysis. Med. Image Anal..

[bib0110] Mackey L. (2008). Deflation Methods for Sparse PCA.

[bib0115] McIntosh A., Bookstein F., Haxby J., Grady C. (1996). Spatial pattern analysis of functional brain images using partial least squares. Neuroimage.

[bib0120] Monteiro J.M., Rao A., Ashburner J., Shawe-Taylor J., Mourão-Miranda J. (2014). Leveraging clinical data to enhance localization of brain atrophy. MLINI 2014 – 4th NIPS Workshop on Machine Learning and Interpretation in Neuroimaging.

[bib0125] Monteiro J.M., Rao A., Ashburner J., Shawe-Taylor J., Mourao-Miranda J. (2015). Multivariate effect ranking via adaptive sparse PLS. International Workshop on Pattern Recognition in NeuroImaging (PRNI).

[bib0130] Mourão-Miranda J., Bokde A.L., Born C., Hampel H., Stetter M. (2005). Classifying brain states and determining the discriminating activation patterns: support vector machine on functional MRI data. Neuroimage.

[bib0135] Nestor P.G., O’Donnell B.F., McCarley R.W., Niznikiewicz M., Barnard J., Shen Z.J., Bookstein F.L., Shenton M.E. (2002). A new statistical method for testing hypotheses of neuropsychological/MRI relationships in schizophrenia: partial least squares analysis. Schizophr. Res..

[bib0140] Nichols T.E., Holmes A.P. (2001). Nonparametric permutation tests for PET functional neuroimaging experiments: a primer with examples. Hum. Brain Mapp..

[bib0145] Nouretdinov I., Costafreda S.G., Gammerman A., Chervonenkis A., Vovk V., Vapnik V., Fu C.H. (2011). Machine learning classification with confidence: application of transductive conformal predictors to MRI-based diagnostic and prognostic markers in depression. Neuroimage.

[bib0150] Nyberg L., McIntosh A.R., Cabeza R., Habib R., Houle S., Tulving E. (1996). General and specific brain regions involved in encoding and retrieval of events: what, where, and when. Proc. Natl. Acad. Sci..

[bib0155] Orrù G., Pettersson-Yeo W., Marquand A.F., Sartori G., Mechelli A. (2012). Using support vector machine to identify imaging biomarkers of neurological and psychiatric disease: a critical review. Neurosci. Biobehav. Rev..

[bib0160] Parkhomenko E., Tritchler D., Beyene J. (2009). Sparse canonical correlation analysis with application to genomic data integration. Stat. Appl. Genet. Mol. Biol..

[bib0165] Price J., Ziolko S.K., Weissfeld L., Klunk W.E., Lu X., Hoge J.A., Meltzer C., Davis S., Lopresti B.J., Holt D.P., DeKosky S.T., Mathis C.A. (2004). Quantitative and statistical analyses of pet imaging studies of amyloid deposition in humans. Nuclear Science Symposium Conference Record, 2004 IEEE, vol. 5.

[bib0170] Rao A., Aljabar P., Rueckert D. (2008). Hierarchical statistical shape analysis and prediction of sub-cortical brain structures. Med. Image Anal..

[bib0175] Rao A., Lee Y., Gass A., Monsch A. (2011). Classification of Alzheimer's Disease from structural MRI using sparse logistic regression with optional spatial regularization. Engineering in Medicine and Biology Society, EMBC, 2011 Annual International Conference of the IEEE.

[bib0180] Tzourio-Mazoyer N., Landeau B., Papathanassiou D., Crivello F., Etard O., Delcroix N., Mazoyer B., Joliot M. (2002). Automated anatomical labeling of activations in SPM using a macroscopic anatomical parcellation of the MNI MRI single-subject brain. Neuroimage.

[bib0185] Waaijenborg S., Verselewel de Witt Hamer P.C., Zwinderman A.H. (2008). Quantifying the association between gene expressions and DNA-markers by penalized canonical correlation analysis. Stat. Appl. Genet. Mol. Biol..

[bib0190] Wegelin J. (2000). A Survey of Partial Least Squares (PLS) Methods, with Emphasis on the Two-block Case.

[bib0195] Witten D.M., Tibshirani R.J. (2009). Extensions of sparse canonical correlation analysis with applications to genomic data. Stat. Appl. Genet. Mol. Biol..

[bib0200] Witten D.M., Tibshirani R., Hastie T. (2009). A penalized matrix decomposition, with applications to sparse principal components and canonical correlation analysis. Biostatistics.

[bib0205] Zou H., Hastie T. (2005). Regularization and variable selection via the elastic net. J. R. Stat. Soc. Ser. B: Stat. Methodol..

