# Supplementary material for: A multiple hold-out framework for Sparse Partial Least Squares
Source: J Neurosci Methods. 2016 Sep 15;271:182–94. doi: 10.1016/j.jneumeth.2016.06.011 (PMC5012894; doi:10.1016/j.jneumeth.2016.06.011)
Supplement: Supplementary file 1 [file mmc1.pdf]

A multiple hold-out framework for Sparse Partial Least Squares

Supplementary material

João M. Monteiro[1,2], Anil Rao[1,2], John Shawe-Taylor[1], and Janaina Mourão-Miranda[1,2]

[1]Department of Computer Science, University College London, London, UK
[2]Max Planck University College London Centre for Computational Psychiatry and Ageing Research, University College London, London, UK

Parameter optimisation

When performing the parameter optimisation step, the average absolute correlation is computed for each parameter combination. Figure 1 shows the values of the mean absolute correlation obtained for the first weight vector pair, in all 10 random splits of the data. As one can see, the surfaces are smooth and fairly consistent across splits, which further supports the reliability of the model. The corresponding results for the second weight vector pair can be seen in Figure 2.

In both multivariate associative effects, the relaxation of the sparsity constraint in the imaging view (c_u) decreased the mean absolute correlation between the projections, which means that both effects are better expressed by a relatively small subset of the image voxels. On the other hand, the optimal constraint on the clinical view (c_v) was in the middle of the parameter range on the first effect, but more relaxed on the second effect (closer to the upper limit of the parameter range). Which means that the first effect will be described by a smaller subset of clinical variables than the second.

This behaviour was not observed if PLS deflation was used instead of projection deflation (Figure 3). In this case, the surfaces were not smooth, the average absolute correlations were lower, and the maximum hyper-parameter combination changes quite a bit when a different split of the data is used. This is consistent with the results that showed a lower average absolute correlation on the hold-out datasets, and lack of statistical significance associated with these weight vectors.

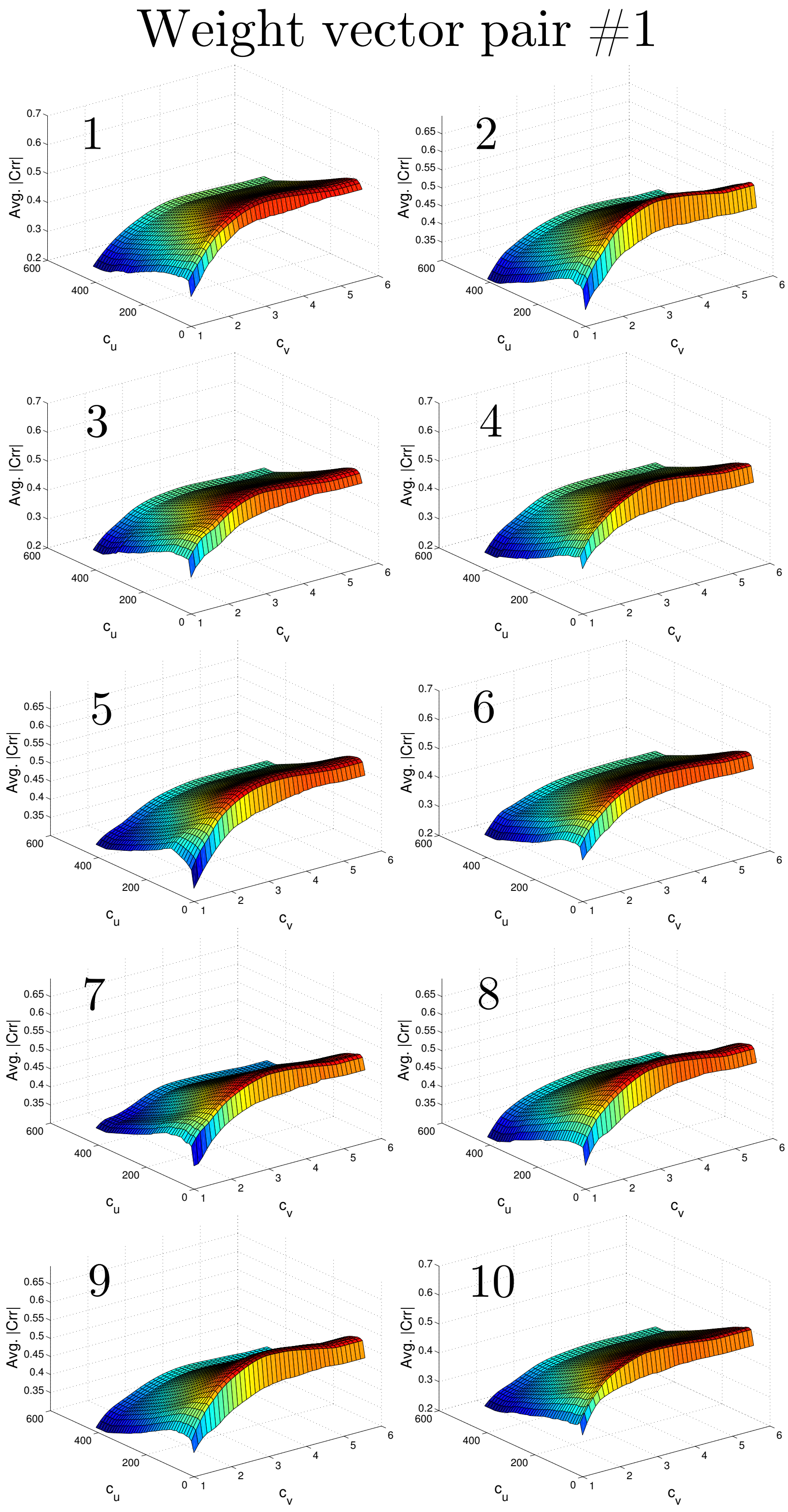

Figure 1: Mean absolute correlation value computed for each split of the data (indicated by the numbers on the top left corners) for the first weight vector pair during the parameter optimisation step.

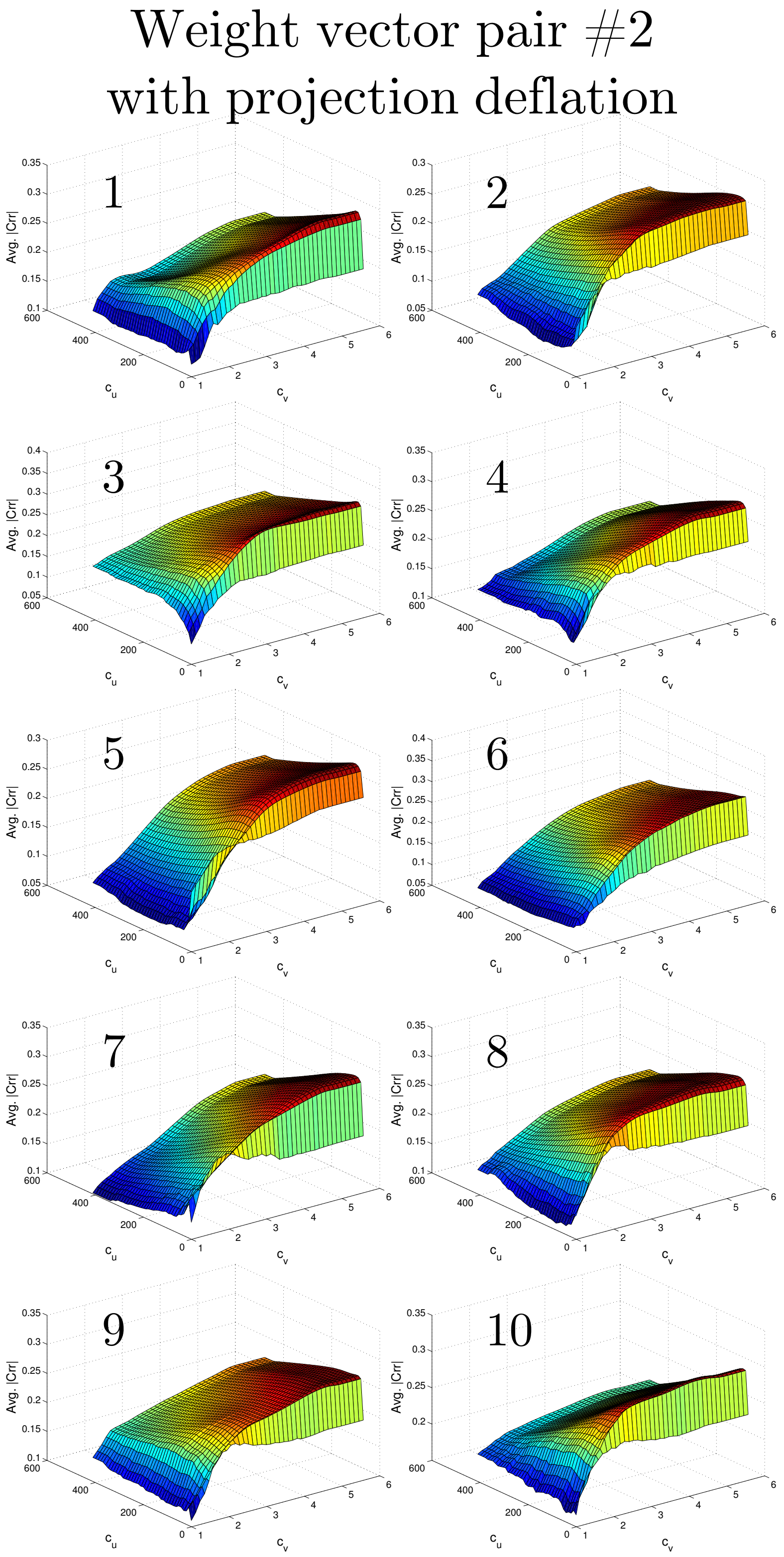

Figure 2: Mean absolute correlation value computed for each split of the data (indicated by the numbers on the top left corners) for the second weight vector pair during the parameter optimisation step.

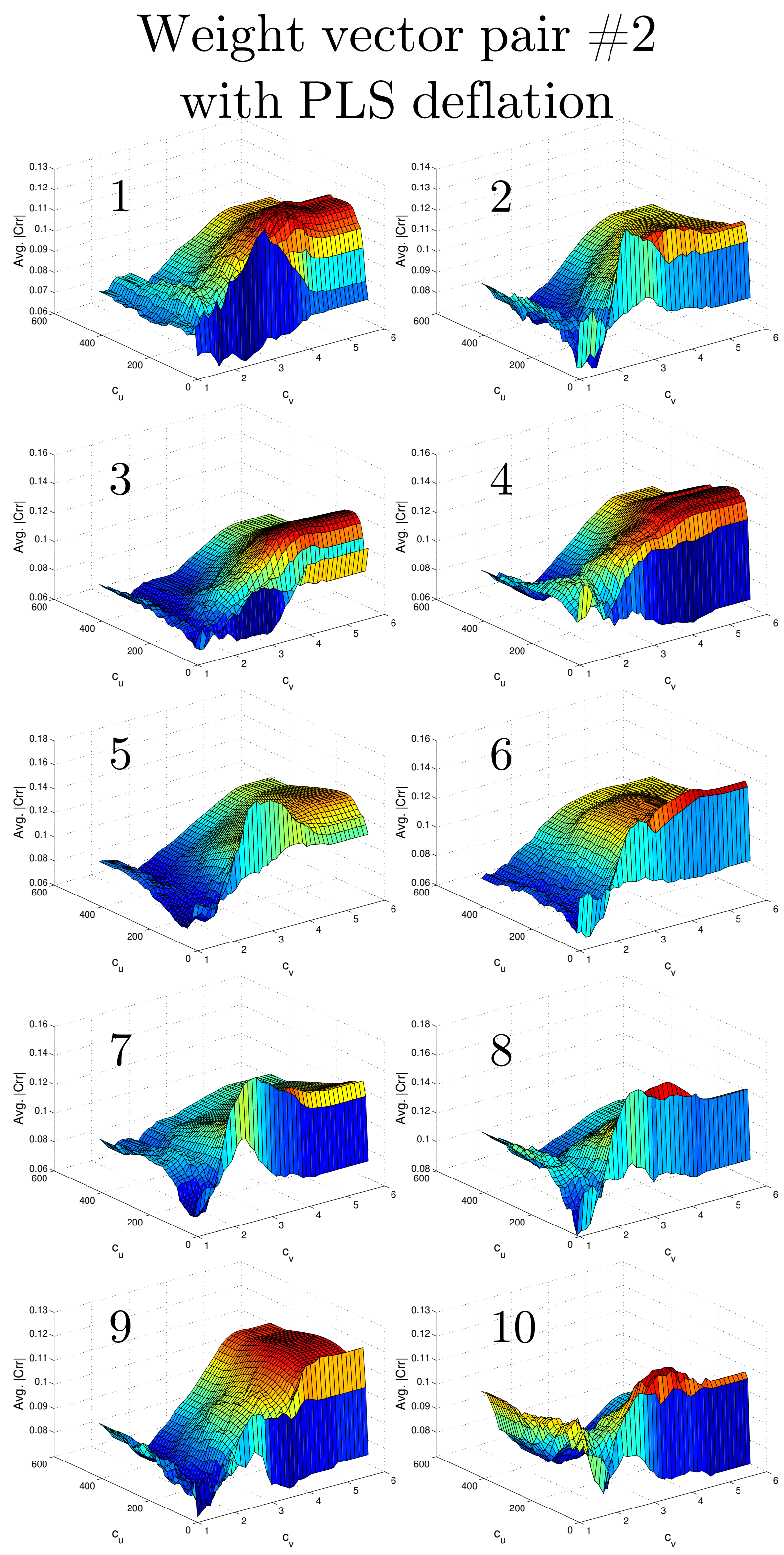

Figure 3: Mean absolute correlation value computed for each split of the data (indicated by the numbers on the top left corners) for the second weight vector pair during the parameter optimisation step.

Atlas regions for each SPLS image weight vector

Table 1: Atlas regions for the first image weight map. Only regions with selected voxels are shown.

**Atlas Region**	**# voxels found**
Amygdala_L	98
Amygdala_R	90
Hippocampus_R	175
Hippocampus_L	152
ParaHippocampal_R	92
ParaHippocampal_L	44
Lingual_L	9
Precuneus_L	2
Precuneus_R	1
Temporal_Pole_Sup_L	1
Fusiform_R	2

Table 2: Atlas regions for the second image weight map. Only regions with selected voxels are shown.

**Atlas Region**	**# voxels found**
Amygdala_L	36
Temporal_Inf_L	292
Hippocampus_L	88
Amygdala_R	11
ParaHippocampal_L	53
Fusiform_L	78
Temporal_Inf_R	64
Hippocampus_R	22
Occipital_Inf_L	12
Temporal_Mid_L	76
Temporal_Mid_R	36
Heschl_R	1
Precuneus_L	17
Angular_R	4
Occipital_Mid_L	14
Temporal_Pole_Mid_L	1
Occipital_Mid_R	3
ParaHippocampal_R	4
Cingulum_Mid_L	5
Angular_L	2
Temporal_Pole_Sup_R	1
Insula_R	2
Precentral_R	2
Insula_L	4
Parietal_Inf_L	1
Lingual_L	2
Parietal_Inf_R	1
Caudate_L	1
Thalamus_L	1
